# Salvage endoscopic resection for perforation site recurrence of colonic polyp

**DOI:** 10.1016/j.vgie.2024.03.011

**Published:** 2024-03-21

**Authors:** Deepak Madhu, Yohei Minato, Hirotsugu Hashimoto, Takuya Takada, Teppei Morikawa, Yoshiaki Kimoto, Shunya Takayanagi, Ken Ohata

**Affiliations:** 1 Department of Gastrointestinal Endoscopy, NTT Medical Center Tokyo, Tokyo, Japan; 2 Department of Diagnostic Pathology, NTT Medical Center Tokyo, Tokyo, Japan; 3 Department of Gastrointestinal Endoscopy, NTT Medical Center Tokyo, Tokyo, Japan

## Abstract

Video 1 Salvage endoscopic resection for perforation site recurrence.

Salvage endoscopic resection for perforation site recurrence.

## Introduction

Recurrence after endoscopic submucosal dissection (ESD) for colonic adenomas can happen rarely.^1^ Salvage ESD has been shown to be feasible in local recurrence of colonic polyps after EMR,^2^ albeit with technical challenges owing to fibrosis. Salvage ESD at a previous ESD-related perforation site is uncommon and is technically challenging due to transmural fibrosis at the site of previous perforation. We report here a salvage endoscopic resection in a patient with a history of ESD-related perforation, who was subsequently noted to have local recurrence of polyp.

## Case

We had previously published the case of a 79-year-old woman with colonic adenoma with high-grade dysplasia (as per World Health Organization classification^3^) who had undergone an ESD for resection.^4^ The ESD was complicated by perforation, which was closed endoscopically with an omental patch and through-the-scope hemostatic clips. Surveillance colonoscopy at 1 year showed local recurrence of the polyp. The initial resection had resulted in indeterminate horizontal and vertical margins, presumably due to the difficulty in resection that resulted from the inadvertant macro-perforation during the procedure. We assume this was the reason for the recurrence. A decision to perform salvage endoscopic resection was made after multidisciplinary evaluation, considering disease characteristics, patient preference, age, and frailty.

## Procedure and Outcomes

**Figure 1:**
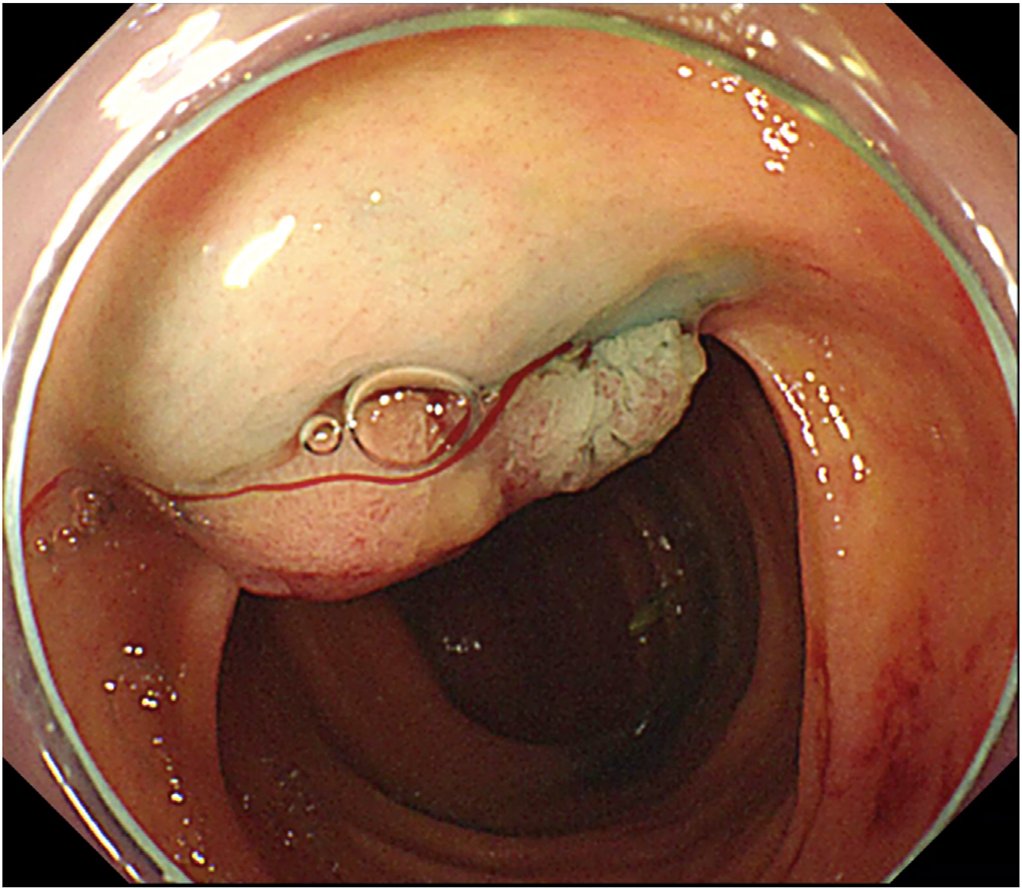
Inadequate lift at the base of the polyp.

**Figure 2:**
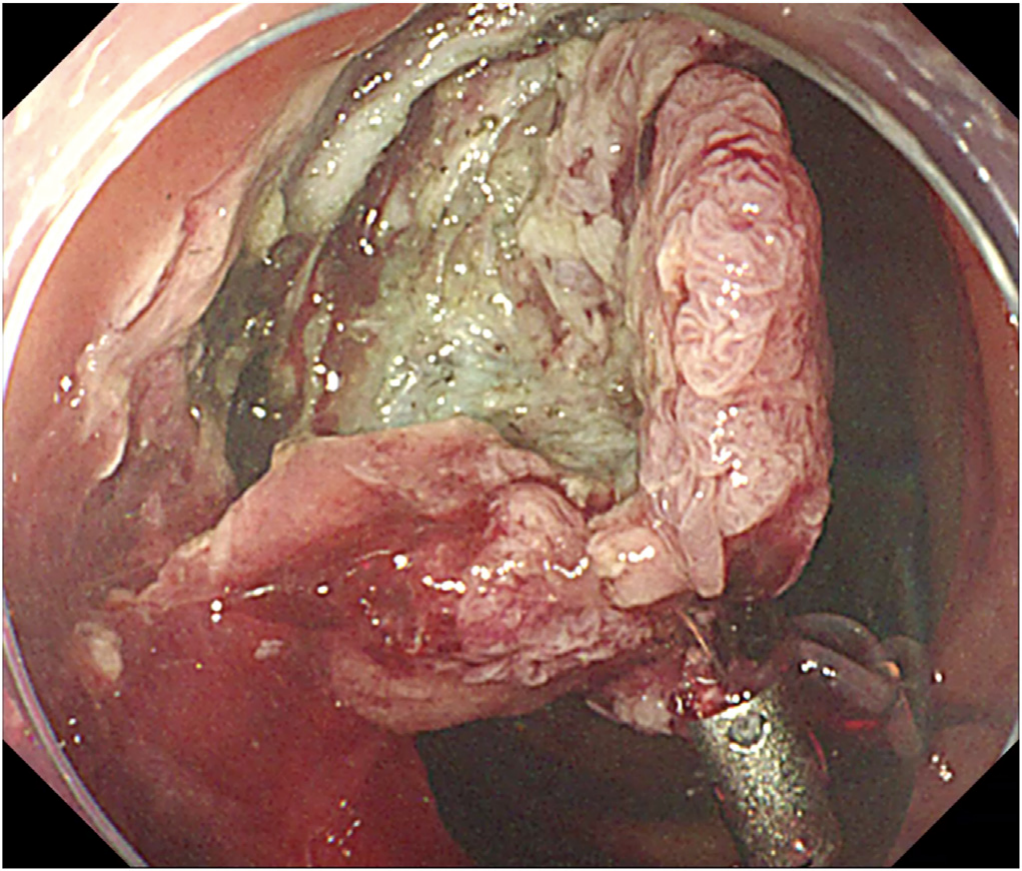
Traction used to widen submucosal space.

**Figure 3:**
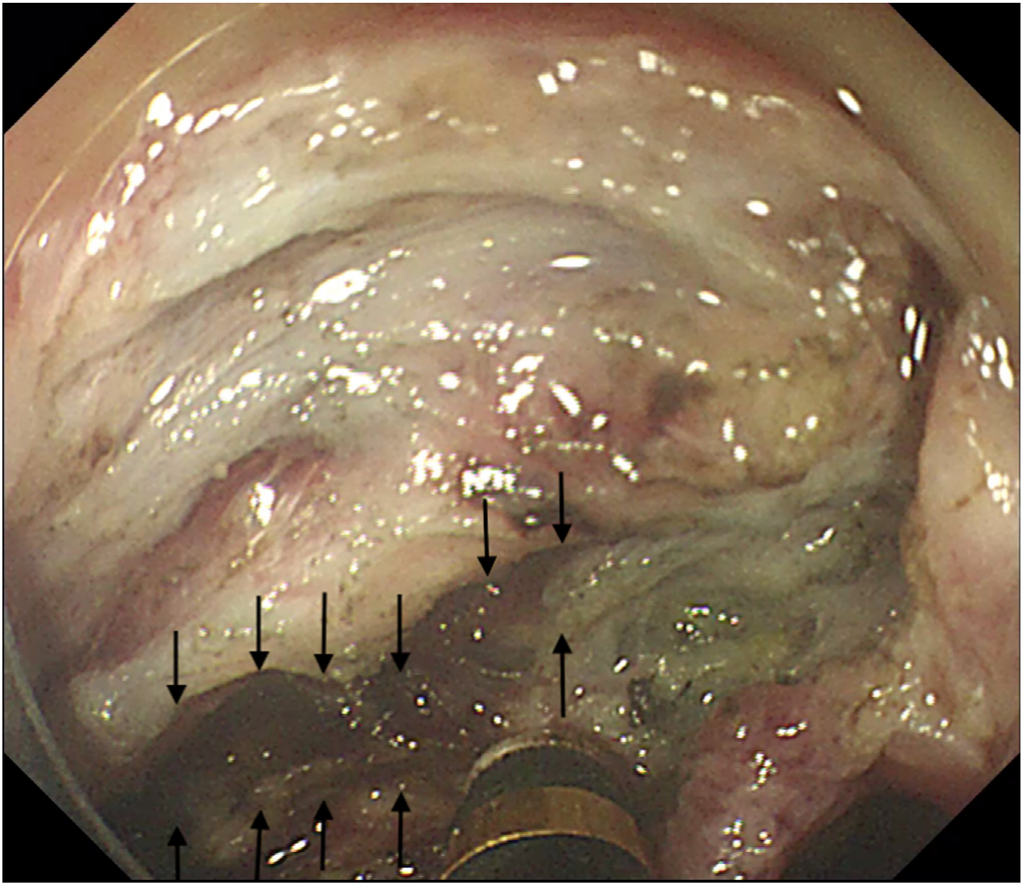
Dissection close to serosa (serosa indicated by *black arrows*).

**Figure 4:**
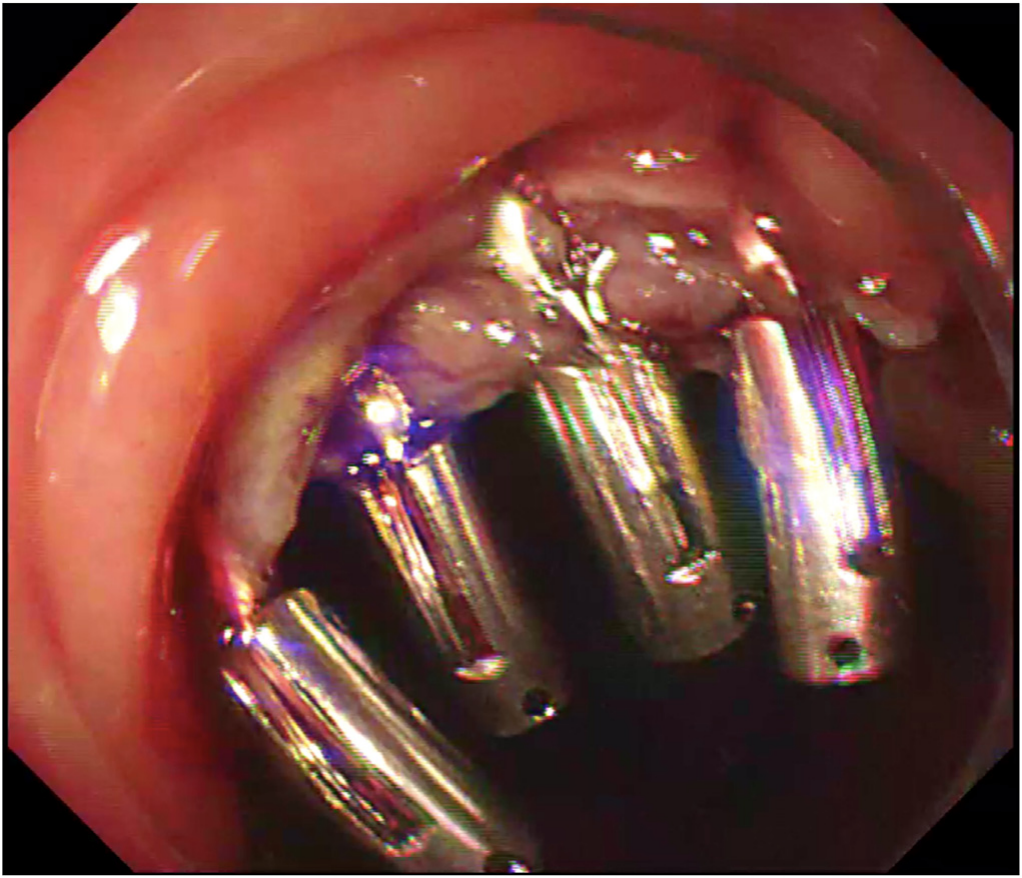
Defect after closure.

The procedure started with submucosal injection (Video 1, available online at www.videogie.org), which showed poor lift at the base of the polyp (Fig. 1), predicting fibrosis. Mucosal incision was performed, but the fibrotic space did not open up wide enough to allow safe dissection. Traction was used to widen the space (Fig. 2). Tunnel method was used for the resection. As dissection proceeded, 2 additional challenges became evident: (1) difficulty in endoscopically discerning the muscle layer separately in parts of the fibrotic space and (2) variable thickness of the fibrotic space, which was very thin in some areas. In areas where the fibrotic space was thinnest, the dissection had to be done close to the serosa (Fig. 3), which served as a guide to the plane of dissection. Once the resection was completed, the area of the defect where serosa was visible endoscopically was closed with through-the-scope hemostatic clips (Fig. 4). There were no postprocedure adverse events, and the patient was discharged uneventfully.

## Discussion

Endoscopic resection of recurrent colonic polyps at a previous site of ESD is technically challenging, owing to fibrosis. Traction^2^ has been shown to be effective in this situation. In our case, the recurrence, which occurred at a site of previous ESD-related perforation, was associated with 2 additional challenges: (1) the absence of a discernible muscle layer endoscopically, to guide the plane of dissection, and (2) very thin areas in the fibrotic space. To tackle these problems, we relied on precise dissection close to the endoscopically visualized serosa, to complete safe resection.

**Figure 5:**
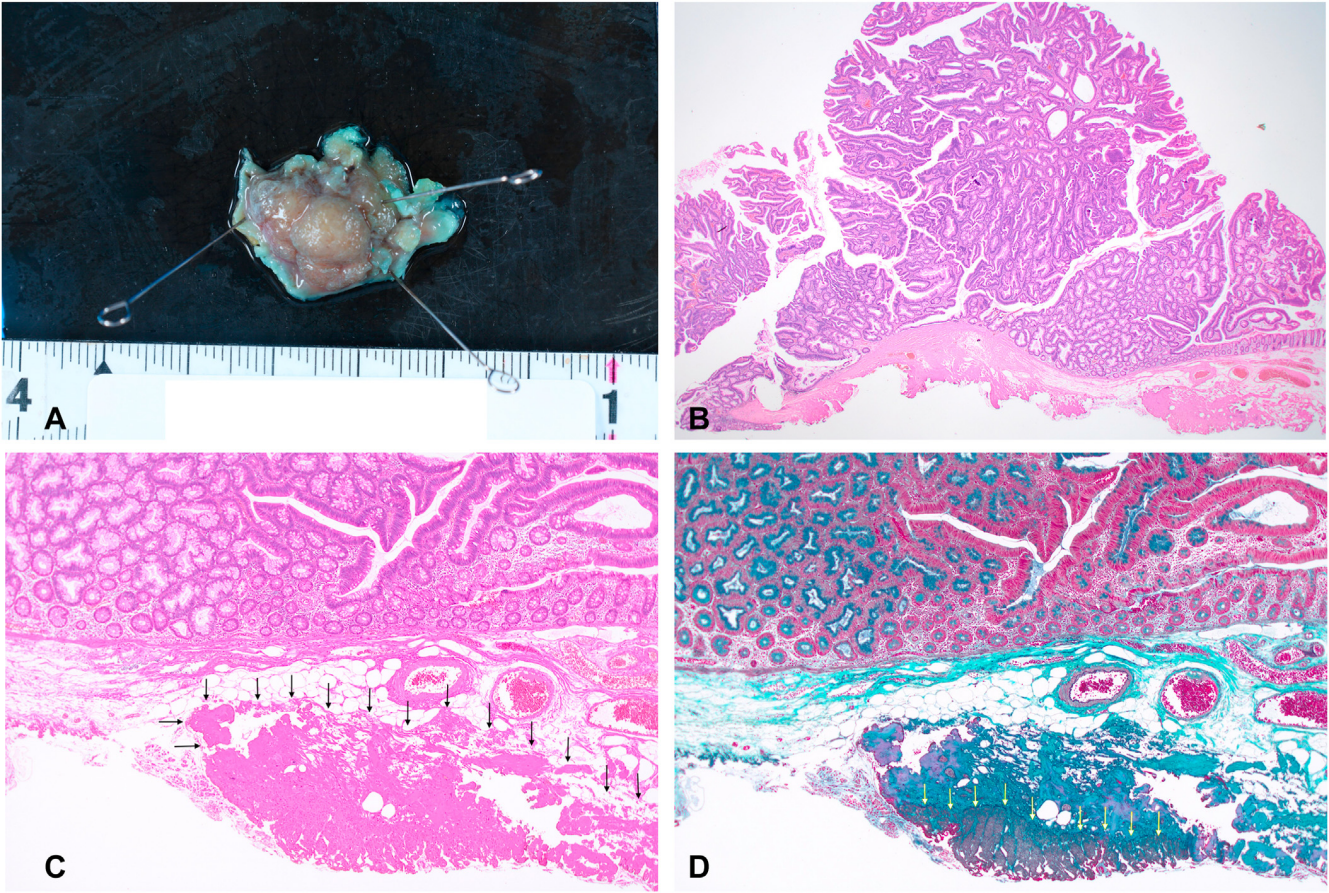
Pathology of the resected specimen. **A,** Macroscopic image of the resected specimen showed 20- × 17-mm-sized sessile polyp (Paris type; 0-Is). **B,** H&E staining with low-power field demonstrates tubular adenoma with high-grade dysplasia (orig. mag. ×1.25, objective lens). **C,** H&E staining demonstrates submucosal fibrosis (*black arrows*) (orig. mag. ×4, objective lens). **D,** Elastica Masson stain demonstrates the presence of muscle fibers within the fibrotic area (*yellow arrows*) (orig. mag. ×4, objective lens).

In our case, the muscle layer was endoscopically difficult to delineate, even though we could notice a few areas of muscularis propria within the fibrotic area in the pathologic assessment of the resected specimen (Fig. 5). We attribute this difficulty to 2 reasons: (1) fibrosis, which involved the submucosa and muscularis propria, and (2) difficulty in obtaining adequate lift with submucosal injection in the fibrotic space, resulting in the absence of differential staining that usually is relied on to identify the plane of dissection during ESD.

## Conclusion

Endoscopic salvage of polyp recurrence at a previous site of perforation could be complicated by fibrosis. A combination of traction, precise dissection, and dissection close to the serosa can be used to tackle these issues to enable safe resection.

## Disclosure

The authors disclosed no financial relationships relevant to this publication.

## Supplementary data

Video 1 Salvage endoscopic resection for perforation site recurrence.
